# Supplementary material for: Concurrent Use of Renal Replacement Therapy during Extracorporeal Membrane Oxygenation Support: A Systematic Review and Meta-Analysis
Source: J Clin Med. 2021 Jan 11;10(2):241. doi: 10.3390/jcm10020241 (PMC7827381; doi:10.3390/jcm10020241)
Supplement: Supplementary file 1 [file jcm-10-00241-s001.pdf]

SUPPLEMENTARY TABLE

Table S1. Joanna Briggs Institute (JBI) checklist for all studies.

Cohort study	Questions											Overall
(1st Author, Year)	1	2	3	4	5	6	7	8	9	10	11	
Allyn, 2018[28]	Yes			Yes	Yes	Yes	Yes		Yes		Yes	7
Antonucci, 2016[29]	Yes	Yes	Yes	Yes	Yes	Yes	Yes		Yes		Yes	9
Baek, 2016[30]	Yes		Yes			Yes	Yes	Yes	Yes		Yes	7
Chen, 2019[31]	Yes	Yes	Yes	Yes	Yes	Yes	Yes	Yes	Yes	Yes	Yes	11
Combes, 2008[32]	Yes			Yes	Yes	Yes	Yes		Yes		Yes	7
Dado, 2020[33]	Yes	Yes	Yes	Yes	Yes	Yes	Yes		Yes		Yes	9
Deatrick, 2020[34]	Yes	Yes	Yes	Yes	Yes	Yes	Yes	Yes	Yes		Yes	10
Devasagayaraj, 2018[35]	Yes	Yes	Yes			Yes	Yes		Yes		Yes	7
Elsharkawy, 2010[36]	Yes			Yes	Yes	Yes	Yes		Yes		Yes	7
Fong, 2020[37]	Yes	Yes	Yes	Yes	Yes	Yes	Yes	Yes	Yes		Yes	10
Haneya, 2015[38]	Yes	Yes	Yes	Yes	Yes	Yes	Yes		Yes		Yes	9
He, 2018[39]	Yes	Yes	Yes	Yes	Yes	Yes	Yes		Yes		Yes	9
Kielstein, 2013[40]	Yes	Yes	Yes	Yes	Yes	Yes	Yes		Yes		Yes	9
Lee SY, 2020[41]	Yes			Yes	Yes	Yes	Yes	Yes	Yes		Yes	8
Luo, 2009[42]	Yes		Yes	Yes	Yes	Yes	Yes	Yes	Yes	Yes	Yes	10
McCanny, 2019[43]	Yes	Yes	Yes	Yes	Yes	Yes	Yes	Yes	Yes		Yes	10
Paek, 2018[44]	Yes	Yes	Yes	Yes	Yes	Yes	Yes	Yes	Yes	Yes	Yes	11
Panholzer, 2017[45]	Yes	Yes	Yes			Yes	Yes		Yes		Yes	7
Schmidt, 2014[46]	Yes	Yes	Yes	Yes	Yes	Yes	Yes	Yes	Yes		Yes	10
Thajudeen, 2015[47]	Yes		Yes	Yes	Yes	Yes	Yes	Yes	Yes	Yes	Yes	10
Unosawa, 2013[48]	Yes			Yes	Yes	Yes	Yes		Yes		Yes	7
Xie, 2020[49]	Yes			Yes	Yes	Yes	Yes	Yes	Yes		Yes	8
Yan, 2010[50]	Yes	Yes	Yes	Yes	Yes	Yes	Yes	Yes	Yes		Yes	10

Case-control Study	Questions										Overall
(1st Author, Year)	1	2	3	4	5	6	7	8	9	10	
Yap, 2003[51]	Yes			Yes	Yes			Yes	Yes	Yes	6

RCT	Questions													Overall
(1st Author, Year)	1	2	3	4	5	6	7	8	9	10	11	12	13	
Li, 2019[52]	Yes	Yes	Yes				Yes	Yes	Yes	Yes	Yes	Yes	Yes	10

SUPPLEMENTARY FIGURES

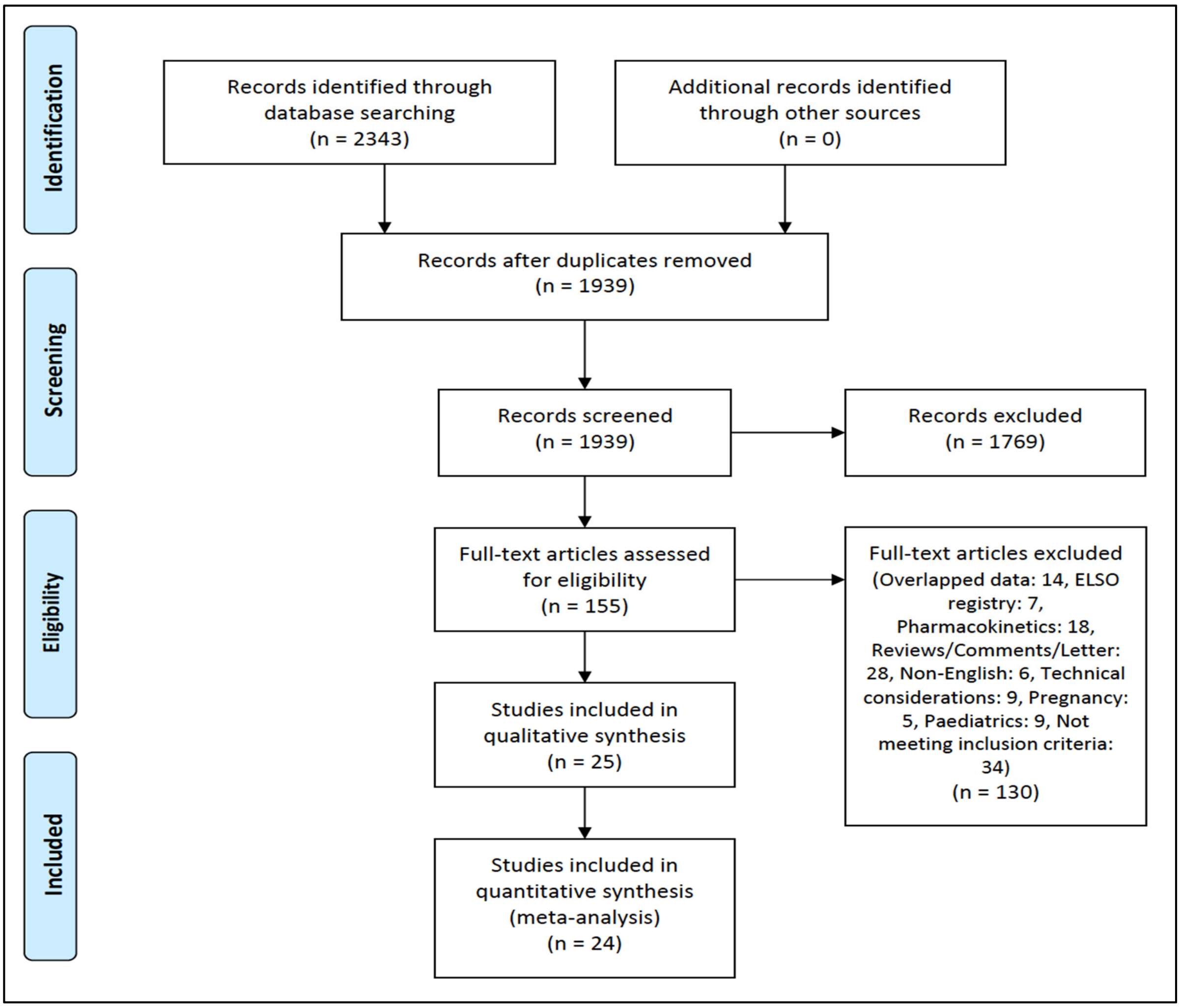

Figure S1. Preferred Reporting Items for Systematic Review and Meta-analyses (PRISMA) flowchart for study selection.

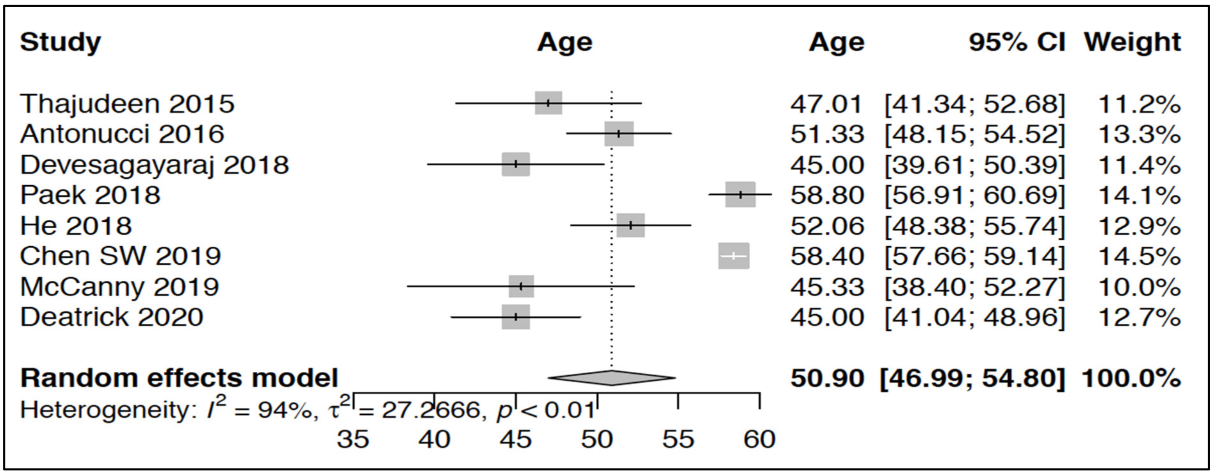

Figure S2. Forest plot showing pooled mean age.

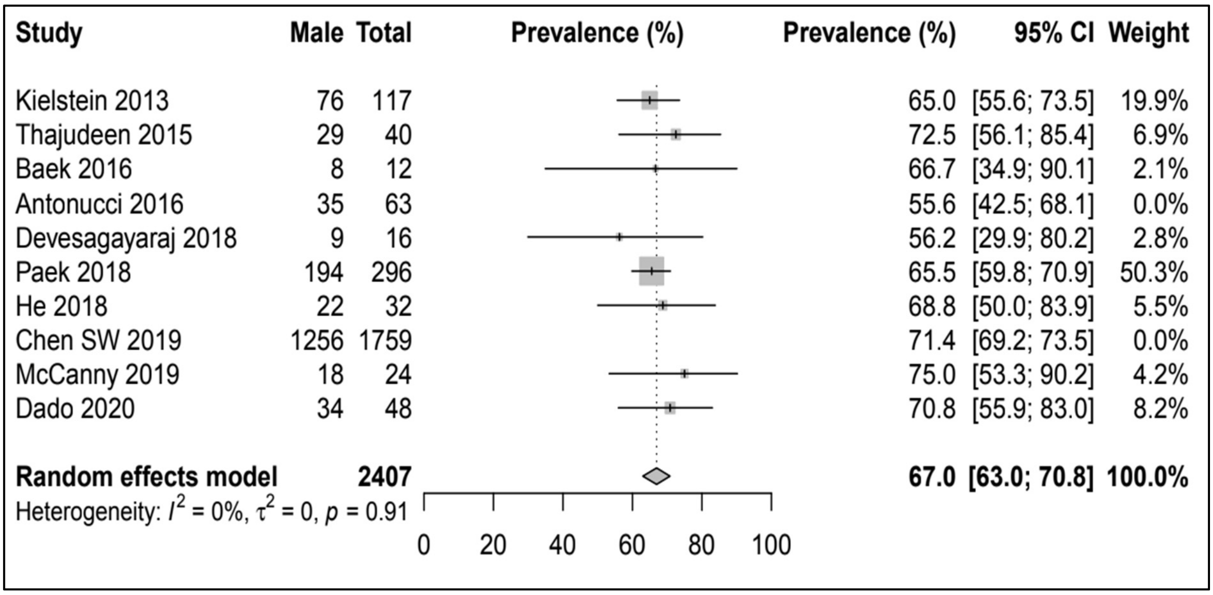

Figure S3. Forest plot showing pooled proportions of males.

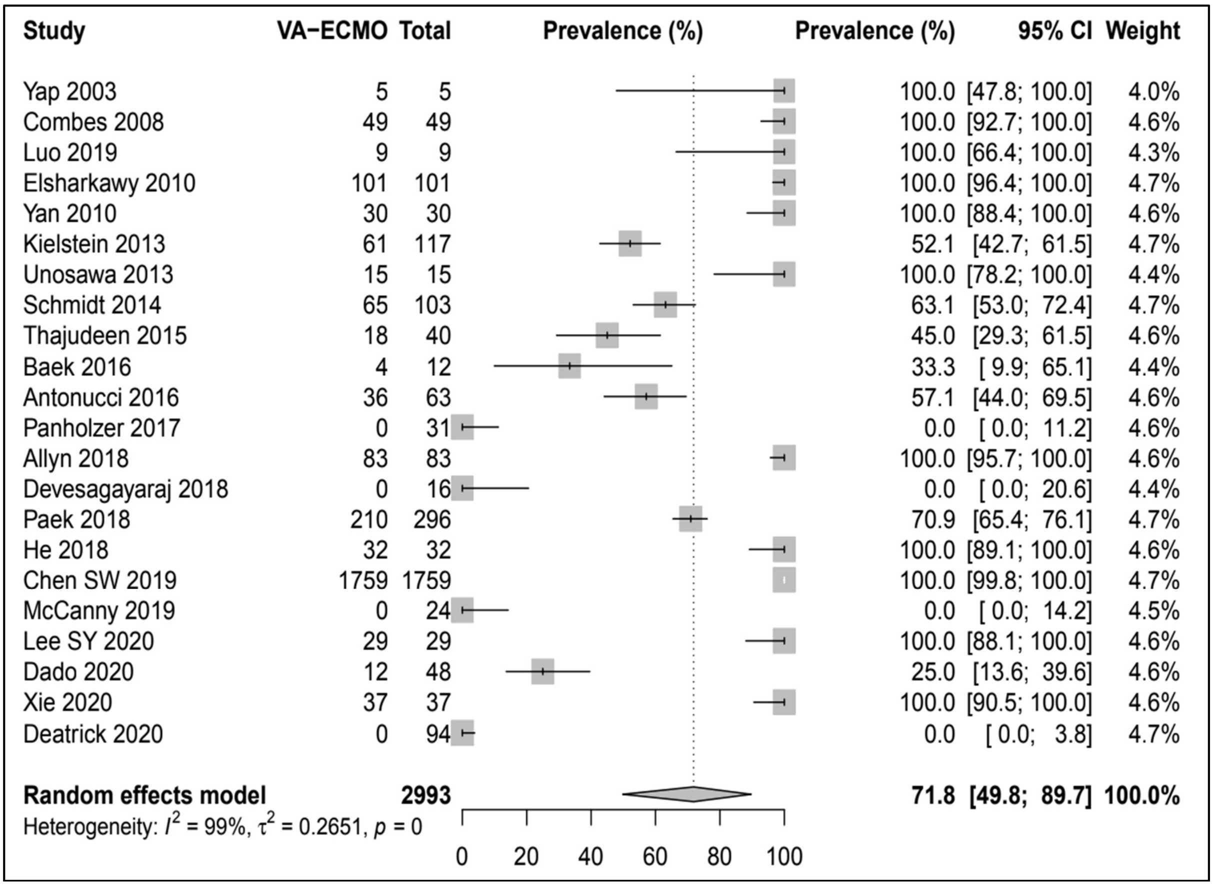

Figure S4. Forest plot showing pooled proportions of concurrent VA ECMO and RRT use.

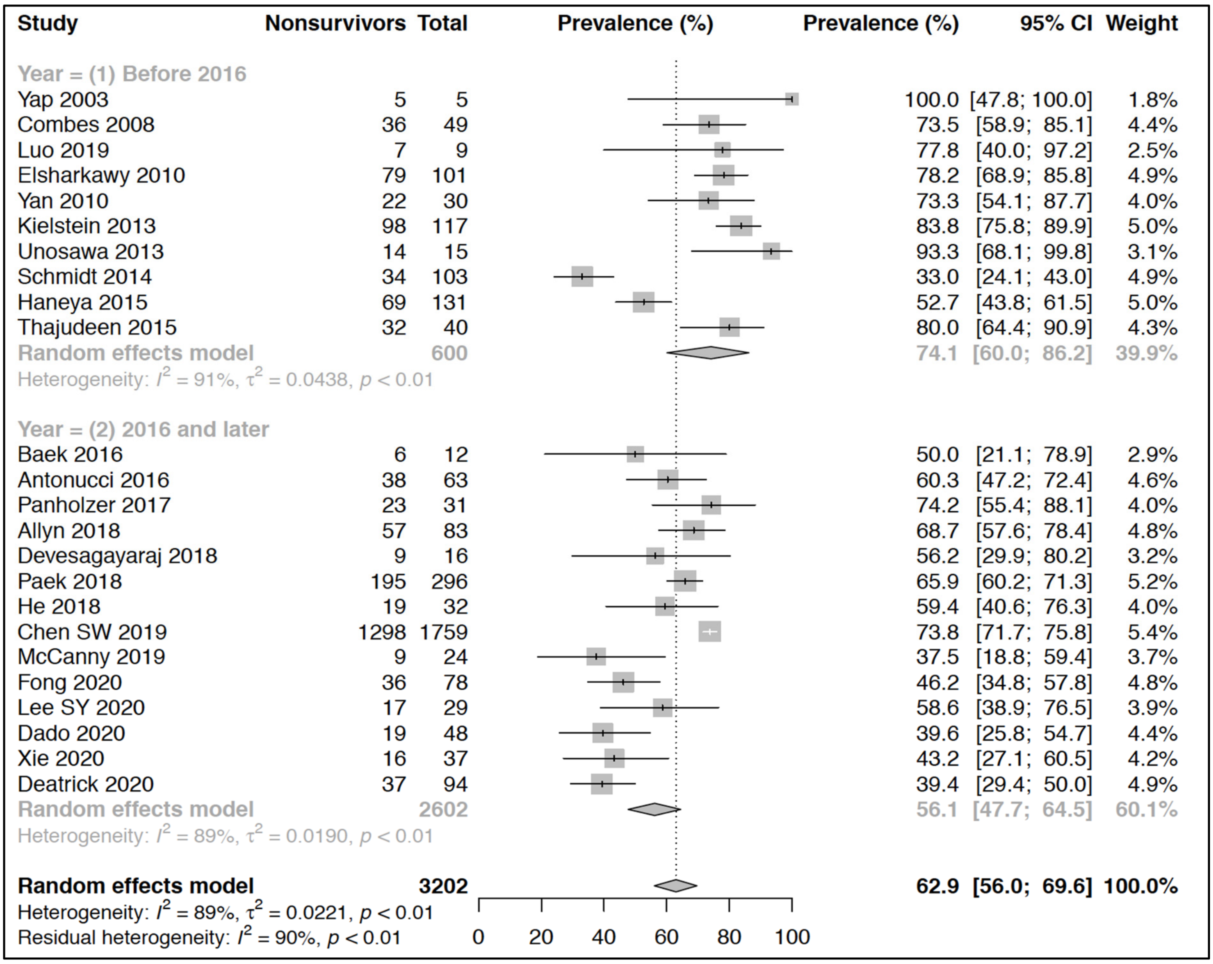

Figure S5. Forest plot showing pooled mortality before and after 2016.

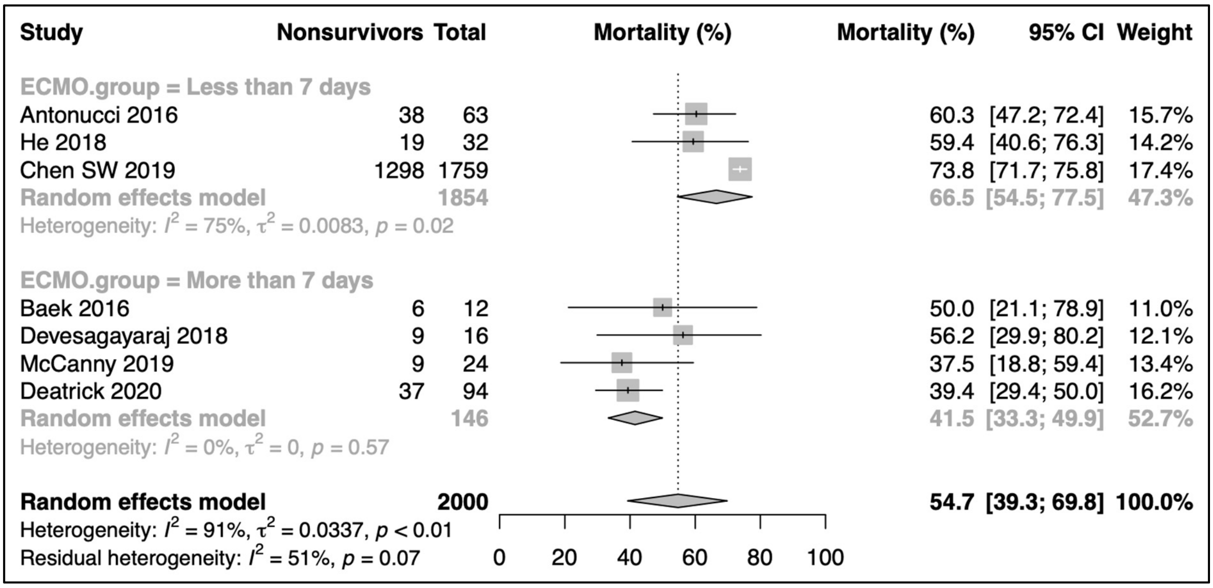

Figure S6. Forest plot showing pooled mortality based on ECMO duration (more and less than 7 days).
